# Serum Potassium and Mortality Risk in Hemodialysis Patients: A Cohort Study

**DOI:** 10.1016/j.xkme.2021.08.013

**Published:** 2021-10-22

**Authors:** Esther N.M. de Rooij, Friedo W. Dekker, Saskia Le Cessie, Ewout J. Hoorn, Johan W. de Fijter, Ellen K. Hoogeveen, Netherlands Cooperative Study on the Adequacy of Dialysis-2 (NECOSAD) Study Group, J.A. Bijlsma, M. Boekhout, W.H. Boer, P.J.M. van der Boog, H.R. Büller, M. van Buren, F.Th. de Charro, C.J. Doorenbos, M.A. van den Dorpel, A. van Es, W.J. Fagel, G.W. Feith, C.W.H. de Fijter, L.A.M. Frenken, W. Grave, J.A.C.A. van Geelen, P.G.G. Gerlag, J.P.M.C. Gorgels, R.M. Huisman, K.J. Jager, K. Jie, W.A.H. Koning-Mulder, M.I. Koolen, T.K. Kremer Hovinga, A.T.J. Lavrijssen, A.J. Luik, J. van der Meulen, K.J. Parlevliet, M.H.M. Raasveld, F.M. van der Sande, M.J.M. Schonck, M.M.J. Schuurmans, C.E.H. Siegert, C.A. Stegeman, P. Stevens, J.G.P. Thijssen, R.M. Valentijn, G.H. Vastenburg, C.A. Verburgh, H.H. Vincent, P.F. Vos

**Affiliations:** 1 Department of Clinical Epidemiology, Leiden University Medical Center, Leiden, The Netherlands; 2 Department of Nephrology, Leiden University Medical Center, Leiden, The Netherlands; 3 Department of Internal Medicine, Division of Nephrology and Transplantation, Erasmus Medical Center, Rotterdam, The Netherlands; 4 Department of Nephrology, Jeroen Bosch Hospital, Den Bosch, The Netherlands

**Keywords:** hemodialysis, hyperkalemia, hypokalemia, mortality, potassium

## Abstract

**Rationale & Objective:**

Both hypo- and hyperkalemia can cause fatal cardiac arrhythmias. Although predialysis serum potassium level is a known modifiable risk factor for death in patients receiving hemodialysis, especially for hypokalemia, this risk may be underestimated. Therefore, we investigated the relationship between predialysis serum potassium level and death in incident hemodialysis patients and whether there is an optimum level.

**Study Design:**

Prospective multicenter cohort study.

**Setting & Participants:**

1,117 incident hemodialysis patients (aged >18 years) from the Netherlands Cooperative Study on the Adequacy of Dialysis-2 study were included and followed from their first hemodialysis treatment until death, transplantation, switch to peritoneal dialysis, or a maximum of 10 years.

**Exposure:**

Predialysis serum potassium levels were obtained every 6 months and divided into 6 categories: ≤4.0 mmol/L, >4.0 mmol/L to ≤4.5 mmol/L, >4.5 mmol/L to ≤5.0 mmol/L, >5.0 mmol/L to ≤5.5 mmol/L (reference), >5.5 mmol/L to ≤6.0 mmol/L, and >6.0 mmol/L.

**Outcomes:**

6-month all-cause mortality.

**Analytical Approach:**

Cox proportional hazards and restricted cubic spline analyses with time-dependent predialysis serum potassium levels were used to calculate the adjusted HRs for death.

**Results:**

At baseline, the mean age of the patients was 63 years (standard deviation, 14 years), 58% were men, 26% smoked, 24% had diabetes, 32% had cardiovascular disease, the mean serum potassium level was 5.0 mmol/L (standard deviation, 0.8 mmol/L), 7% had a low subjective global assessment score, and the median residual kidney function was 3.5 mL/min/1.73 m^2^ (IQR, 1.4-4.8 mL/min/1.73 m^2^). During the 10-year follow-up, 555 (50%) deaths were observed. Multivariable adjusted HRs for death according to the 6 potassium categories were as follows: 1.42 (95% CI, 1.01-1.99), 1.09 (95% CI, 0.82-1.45), 1.21 (95% CI, 0.94-1.56), 1 (reference), 0.95 (95% CI, 0.71-1.28), and 1.32 (95% CI, 0.97-1.81).

**Limitations:**

Shorter intervals between potassium measurements would have allowed for more precise mortality risk estimations.

**Conclusions:**

We found a U-shaped relationship between serum potassium level and death in incident hemodialysis patients. A low predialysis serum potassium level was associated with a 1.4-fold stronger risk of death than the optimal level of approximately 5.1 mmol/L. These results may imply the cautious use of potassium-lowering therapy and a potassium-restricted diet in patients receiving hemodialysis.

## Graphical abstract

**Figure:**
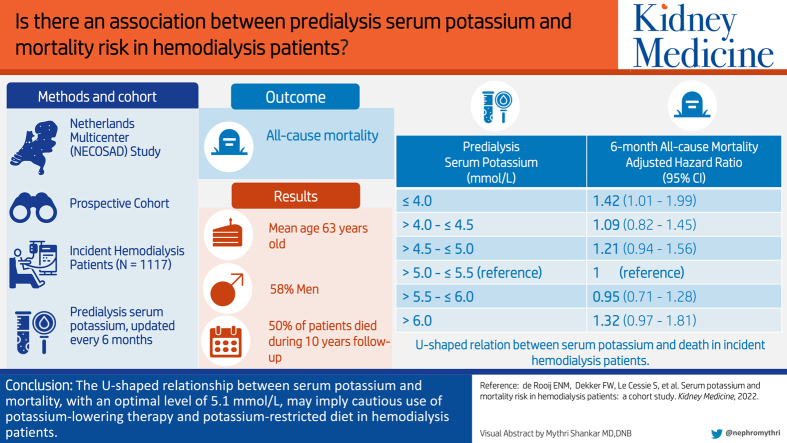

**Plain-Language Summary**

Both high and low serum potassium levels can cause fatal heart arrhythmias. In patients receiving hemodialysis, the mortality risk of low serum potassium levels, in particular, may have been underestimated. Therefore, we investigated the relationship between predialysis serum potassium level and 6-month mortality in 1,117 patients receiving hemodialysis during 10 years of follow-up. In our analysis, we adjusted for the potential confounders of age, sex, diabetes, cardiovascular disease, smoking, residual kidney function, and nutritional state. We found that low (≤4.0 mmol/L) and high (>6.0 mmol/L) predialysis serum potassium levels are associated with a 1.4- and 1.3-fold, respectively, stronger risk of death than the optimal serum potassium level of approximately 5.1 mmol/L. These results may imply the cautious use of potassium-lowering therapy and a potassium-restricted diet in patients receiving hemodialysis.

Accumulating evidence points to the adverse impact of dyskalemia on life expectancy in patients receiving hemodialysis.^1^ Both hypo- and hyperkalemia can cause potentially fatal cardiac arrhythmias and sudden death.^2^ Potassium homeostasis is mainly regulated by the kidneys, responsible for excreting 90% of the dietary potassium intake. Patients receiving hemodialysis rely mainly on potassium removal during each dialysis session.^2^ Hyperkalemia, defined as a serum potassium level of ≥5.5 mmol/L, is a common electrolyte disorder occurring in 12%-20% of patients receiving hemodialysis.^3^, ^4^, ^5^, ^6^ Nonadherence to dietary potassium restrictions and metabolic acidosis increase the risk of hyperkalemia. Relatively little attention is paid to hypokalemia and a low potassium level, defined as serum potassium levels of <3.5 mmol/L and <4.0 mmol/L, respectively, occurring in 2% and 13% of patients receiving hemodialysis, respectively.^3^^,^^5^^,^^6^ Malnourishment, metabolic alkalosis, potassium binders, and low potassium dialysate are risk factors for hypokalemia.^1^^,^^5^ In addition, patients receiving hemodialysis have low intracellular potassium concentrations, despite the tendency of hyperkalemia development in them. Total body potassium levels can be up to 10% lower in patients receiving hemodialysis than in controls and is associated with an increased risk of hypertension, cardiovascular disease, and death.^7^^,^^8^

The optimal predialysis serum potassium level is unknown. There are no randomized controlled trials that have examined the target predialysis potassium level with regard to long-term outcomes. Therefore, we have to rely on prospective cohort and registry studies. In the general population, serum potassium levels between 3.5 and 5.0 mmol/L are considered to be within the normal range. In patients with chronic kidney disease, the optimal range is between 4.0 and 4.5 mmol/L.^9^ An important limitation to previous studies investigating the relationship between serum potassium levels and death on hemodialysis is the inclusion of mainly prevalent instead of incident patients, which may have resulted in survivor bias.^10^ Furthermore, until now, cohort studies have estimated the risk of low serum potassium levels in comparison with relatively low levels (4.0-4.5 mmol/L) as a reference category and, therefore, most likely underestimated the mortality risk (hazard ratios [HRs], 1.03-1.14) related to hypokalemia.^4^^,^^11^^,^^12^

Low predialysis serum potassium level is a potentially modifiable risk factor; however, its adverse effect on death in patients receiving hemodialysis may be underestimated. Therefore, we studied the relationship between serum potassium levels and death in a prospective cohort of incident hemodialysis patients and investigated whether there is an optimum level to pursue. Results from this study may inform future guidelines for patients receiving hemodialysis.

## Methods

### Study Design and Population

The Netherlands Cooperative Study on the Adequacy of Dialysis-2 is a prospective multicenter cohort study of patients, aged >18 years, with incident end-stage renal disease, starting with their first dialysis treatment, as previously described in detail.^13^ Briefly, enrollment occurred between 1997 and 2007 at 38 dialysis centers throughout the Netherlands. The maximum follow-up was 10 years after the start of hemodialysis, with the latest follow-up date on January 1, 2018. The institutional review board of the Academic Medical Hospital, Amsterdam, the Netherlands approved the study (approval number, MEC95/226a) and the institutional review boards of all participating hospitals confirmed this by an additional local approval. All patients gave written informed consent. Follow-up visits were scheduled at 3 months after the start of dialysis therapy, at 6 months, and subsequently at intervals of 6 months. All centers predominantly used a dialysate potassium concentration of 2 mmol/L and, if indicated, 3 mmol/L. Baseline was defined as 3 months after the start of hemodialysis treatment, when the patients’ fluid and metabolic conditions had stabilized. Dates of mortality were immediately reported during follow-up and ascertained by a nephrologist.

The cohort comprised 1,117 patients receiving hemodialysis, without previous kidney replacement therapy, 3 months after starting dialysis. Survival time was defined as the number of days between 3 months after the start of the hemodialysis treatment (baseline) and the date of death, the date of censoring due to loss to follow-up, kidney transplantation, transfer to a nonparticipating dialysis center, a switch to peritoneal dialysis treatment, or the end of the follow-up.

### Data Collection

Demographic and clinical data such as age, sex, ethnicity, primary kidney disease, current smoking, medication, a history of diabetes, cardiovascular disease, malignancy or chronic lung disease, Subjective Global Assessment (SGA), blood pressure, blood, and 24-hour urine samples were collected at the start of dialysis treatment and at all visits until the end of follow-up. Primary kidney disease was classified according to the codes of the European Renal Association–European Dialysis and Transplant Association.^13^ We grouped patients into 4 classes of primary kidney disease: diabetic nephropathy, glomerulonephritis, renal vascular disease, and other kidney diseases. A history of diabetes was defined based on diabetes mellitus registered as a comorbid condition or diabetic nephropathy as primary kidney disease. Current smoking was defined as current cigarette smokers, including those who quit smoking in the past 3 months. Cardiovascular disease was defined as any history of a cerebral vascular accident, a myocardial infarction, or peripheral vascular disease.

For blood pressure, the mean systolic and diastolic blood pressure values prior to dialysis over the previous 2 weeks were calculated. The nutritional state was measured using a 1-7 score on SGA, with scores of 6-7 indicating normal protein-energy wasting, scores of 4-5 indicating moderate protein-energy wasting, and scores of 1-3 indicating severe protein-energy wasting.^14^

Serum potassium, albumin, creatinine, and urea levels were measured during the prelude to dialysis and after the long dialysis interval. In addition, urea and creatinine levels were also measured in the urine. Residual kidney function was estimated using combined urea and creatinine clearance and corrected for body surface (mL/min/1.73 m^2^).

### Statistical Analysis

Variables are presented as means ± standard deviations (SDs), median (interquartile range), or numbers (proportions) where appropriate and according to 6 predefined baseline predialysis serum potassium level categories: ≤4 mmol/L, >4 mmol/L to ≤4.5 mmol/L, >4.5 mmol/L to ≤5.0 mmol/L, >5.0 mmol/L to ≤5.5 mmol/L, >5.5 mmol/L to ≤6.0 mmol/L, and >6.0 mmol/L.

We assessed the relationship between predialysis serum potassium level and all-cause mortality during 10 years of follow-up using several methods. In all analyses, survival was measured from 3 months (baseline) after the start of hemodialysis. The survival probabilities for the 6 predialysis serum potassium categories at baseline were visualized using Kaplan-Meier curves for the first 10 years of follow-up.

For all following analyses, the predialysis serum potassium level was included as a time-dependent variable and updated at a 6-month interval after the start of the first hemodialysis treatment. First, we used life tables to calculate absolute mortality rates during the follow-up within each of the 6 time-dependent predialysis serum potassium level categories. Second, we used a time-dependent Cox proportional hazards model to calculate crude and multivariable adjusted HRs for 6-month all-cause mortality during 10 years of follow-up. As normal serum potassium levels vary from 3.5 to 5.5 mmol/L and no optimum level has been definitively recommended within that range, the predialysis serum potassium category (>5.0 to ≤5.5 mmol/L) with the lowest mortality rate in our cubic spline analysis was considered the reference category.^15^ Analyses were adjusted for potential confounders measured at baseline: age, sex, current smoking, history of diabetes, history of cardiovascular disease, residual kidney function, and SGA score (full model). We did not control for time-varying confounding because later values of time-dependent confounders may be influenced by earlier levels of predialysis serum potassium. Therefore, adjustments for such time-dependent variables could have introduced bias.^16^ Third, the continuous relationship between time-dependent predialysis serum potassium level and mortality was explored by modeling a 4-knot restricted cubic spline with 95% confidence intervals (CIs) adjusted for the previously mentioned confounders. The knots were chosen at the 5th, 35th, 65th, and 95th percentiles of the predialysis serum potassium level distribution.^17^

We performed 4 sensitivity analyses. First, we repeated our full time-dependent Cox proportional hazards model with additional adjustments for serum phosphate level, serum bicarbonate level, serum albumin level, and normalized protein catabolic rate to assess any residual confounding by nutritional state. Second, we performed time-dependent Cox proportional hazard analyses considering as a secondary outcome cardiac death due to dyskalemia, cardiac arrest, myocardial infarction, and sudden death. We did not include cardiac death in our primary analysis as specific causes of death are more likely to be misclassified. Third, we repeated our analyses without censoring for patients switching to peritoneal dialysis. In the primary analyses, we censored patients from the moment they switched from hemodialysis to peritoneal dialysis, since we aimed to investigate only the effect of the predialysis serum potassium level on the mortality risk in patients receiving hemodialysis. However, this might have resulted in informative censoring, since patients switching to peritoneal dialysis are often in better or worse clinical condition than the general population receiving hemodialysis. Fourth, we repeated our Cox proportional hazards model using the baseline predialysis serum potassium level category as a fixed variable to calculate the HRs for 10-year all-cause mortality and cardiac death. This enabled us to evaluate whether the relationship between predialysis serum potassium level and all-cause mortality is indeed a short-term effect that can be better estimated using a time-dependent analysis.

We assumed missing values to be missing at random. Missing data were handled using 2 different strategies. For missing predialysis serum potassium levels at baseline (n = 9; 1%), we carried the next observed predialysis serum potassium level backward, and for the missing values during the 10-year follow-up (n = 869; 13%), we carried the last observed predialysis serum potassium level forward. For the following missing baseline data—smoking (n = 8; 1%), history of diabetes (n = 14; 1%) or history of cardiovascular disease (n = 14; 1%), residual kidney function (n = 259; 23%), and SGA score (n = 82; 7%), we used multiple imputations to avoid bias and maintain power, using 10 imputations, and including all relevant baseline variables and the outcome in the model.^18^

In the proportional hazards regression models, the proportionality assumption for each covariate was checked by adding a product term between that covariate and the logarithm of follow-up time. All analyses were performed using SPSS 23.0 (International Business Machines Corporation) and R version 3.5.1 (R Core Team).

## Results

### Baseline Characteristics

Of all Netherlands Cooperative Study on the Adequacy of Dialysis-2 study participants, 1,117 patients receiving hemodialysis survived the first 3 months after starting dialysis and were therefore included in the analysis. At baseline, the mean age of the study cohort was 63 years (SD, 14 years), 58% were men, 26% were current smokers, 24% had a history of diabetes, 32% had a history of cardiovascular disease, 7% had a low SGA score, and the median residual kidney function was 3.5 mL/min/1.73 m^2^ (interquartile range, 1.4-4.8 mL/min/1.73 m^2^). The mean predialysis serum potassium level was 5.0 mmol/L (SD, 0.8 mmol/L). The prevalence of the 6 predialysis serum potassium level categories of ≤4.0 mmol/L, >4.0 mmol/L to ≤4.5 mmol/L, >4.5 mmol/L to ≤5.0 mmol/L, >5.0 mmol/L to ≤5.5 mmol/L (reference), >5.5 mmol/L to ≤6.0 mmol/L, and >6.0 mmol/L were 10%, 19%, 26%, 22%, 15%, and 8%, respectively. Mean predialysis serum potassium levels in the lowest (SD, ≤4.0 mmol/L) and highest (SD, >6.0 mmol/L) potassium categories were 3.7 mmol/L (SD, 0.3 mmol/L) and 6.5 mmol/L (SD, 0.3 mmol/L), respectively. Table 1 presents the baseline characteristics for all patients and according to the 6 predialysis serum potassium level categories. Compared with the reference category (>5.0 to ≤5.5 mmol/L), patients in the lowest potassium level category were older, smoked less often, and had lower SGA scores, whereas those in the highest predialysis serum potassium level category were younger and had lower residual kidney functions. Phosphate levels increased with higher potassium level categories, whereas bicarbonate levels decreased.

**Table 1:** Baseline Characteristics of 1,117 Incident Hemodialysis Patients, Overall and According to 6 Predialysis Serum Potassium Level Categories

Characteristic	All Patients	Predialysis Serum Potassium Level Category, mmol/L					
		≤4.0	>4.0 to ≤4.5	>4.5 to ≤5.0	>5.0 to ≤5.5	>5.5 to ≤6.0	>6.0
Total, n (%)	1,117	117 (10)	208 (19)	287 (26)	241 (22)	170 (15)	94 (8)
Age, y	63 (14)	66 (13)	67 (11)	64 (14)	61 (14)	62 (14)	59 (15)
Men, n (%)	649 (58)	70 (60)	117 (56)	164 (57)	147 (61)	94 (55)	57 (61)
Ethnicity, n (%)							
White	1,030 (92)	108 (92)	196 (94)	265 (92)	222 (92)	158 (93)	81 (86)
Black	25 (2)	4 (3)	3 (2)	4 (1)	8 (3)	3 (2)	3 (3)
Asian	60 (6)	5 (4)	9 (4)	18 (6)	11 (5)	9 (5)	8 (9)
Other	2 (0)	0 (0)	0 (0)	0 (0)	0 (0)	0 (0)	2 (2)
BMI, kg/m^2^	24.7 (4.4)	24.5 (4.4)	25.0 (4.8)	25.2 (4.5)	24.1 (3.9)	24.8 (4.3)	24.1 (4.2)
SGA, n (%)							
1-3; severe PEW	70 (7)	17 (15)	17 (9)	14 (5)	8 (4)	9 (6)	5 (6)
4-5; moderate PEW	275 (26)	31 (28)	65 (34)	63 (23)	54 (24)	40 (25)	22 (27)
6-7; normal PEW	690 (67)	62 (57)	109 (57)	193 (72)	159 (72)	112 (70)	55 (67)
Current smoking, n (%)	288 (26)	26 (22)	54 (26)	68 (24)	74 (31)	38 (22)	28 (30)
Primary kidney disease, n (%)							
Diabetic nephropathy	176 (16)	18 (16)	35 (17)	42 (15)	37 (15)	30 (18)	14 (15)
Glomerulonephritis	113 (10)	10 (9)	12 (6)	30 (11)	24 (10)	21 (13)	16 (17)
Renal vascular disease	245 (22)	24 (21)	55 (27)	62 (22)	52 (22)	30 (18)	22 (24)
Other	574 (52)	64 (55)	104 (51)	153 (53)	128 (53)	85 (51)	40 (44)
Kt/V,^a^ wk	3.5 (2.8-4.0)	3.7 (3.2-4.3)	3.6 (2.8-4.1)	3.4 (2.8-4.0)	3.3 (2.7-3.8)	3.3 (2.7-3.9)	3.3 (2.7-3.9)
Residual eGFR,^a^^,^^b^ mL/min/1.73 m^2^	3.5 (1.4-4.8)	3.5 (2.0-6.3)	3.8 (2.0-5.7)	3.5 (1.6-5.1)	3.0 (1.6-4.1)	2.3 (1.0-3.8)	1.5 (0.5-3.4)
Dialysis frequency, n (%)							
1 d/wk	10 (1)	1 (1)	2 (1)	6 (2)	0 (0)	1 (1)	0 (0)
2 d/wk	430 (39)	44 (38)	81 (39)	120 (42)	94 (39)	56 (34)	35 (37)
3 d/wk	671 (60)	72 (62)	122 (59)	161 (56)	147 (61)	110 (66)	59 (63)
Systolic BP,^c^ mm Hg	148 (19)	144 (21)	146 (19)	150 (18)	148 (20)	148 (20)	149 (17)
Diastolic BP,^c^ mm Hg	80 (10)	79 (10)	78 (10)	80 (9)	81 (10)	80 (11)	82 (10)
History of diabetes, n (%)	260 (24)	31 (27)	55 (27)	66 (23)	51 (21)	37 (22)	20 (22)
History of cardiovascular disease,^d^ n (%)	354 (32)	44 (38)	79 (38)	92 (32)	70 (29)	50 (29)	19 (20)
History of chronic lung disease, n (%)	98 (9)	11 (10)	23 (11)	22 (8)	21 (9)	13 (8)	8 (9)
History of malignancy, n (%)	131 (12)	13 (11)	29 (14)	31 (11)	26 (11)	24 (15)	8 (9)
Antihypertensives, n (%)	884 (80)	93 (80)	160 (77)	233 (82)	195 (81)	133 (79)	70 (78)
Insulin, n (%)	149 (13)	18 (16)	33 (16)	31 (11)	31 (13)	22 (13)	14 (15)
Lipid-lowering drugs, n (%)	284 (26)	30 (26)	62 (30)	77 (27)	59 (25)	36 (22)	20 (22)
Potassium, mmol/L	5.0 (0.8)	3.7 (0.3)	4.3 (0.1)	4.8 (0.1)	5.3 (0.1)	5.7 (0.1)	6.5 (0.3)
Hemoglobin, mmol/L	6.7 (0.9)	6.6 (1.1)	6.6 (1.0)	6.7 (0.8)	6.7 (0.8)	6.7 (0.9)	6.5 (0.8)
Bicarbonate, mmol/L	22 (3)	24 (3)	23 (3)	22 (3)	22 (3)	21 (4)	21 (3)
Cholesterol, mmol/L	4.7 (1.2)	4.8 (1.4)	4.9 (1.3)	4.6 (1.2)	4.6 (1.1)	4.9 (1.3)	4.7 (1.0)
Phosphate, mmol/L	1.9 (0.6)	1.6 (0.6)	1.7 (0.5)	1.8 (0.5)	1.9 (0.5)	2.0 (0.6)	2.2 (0.7)
Albumin, g/L	35 (6)	35 (6)	36 (6)	36 (6)	35 (6)	36 (6)	36 (6)
nPCR,^e^ g/kg/d	1.0 (0.2)	1.0 (0.2)	1.0 (0.3)	1.0 (0.2)	1.0 (0.2)	1.0 (0.2)	1.0 (0.2)

*Note:* Continuous data are expressed as the mean (± standard deviation), unless otherwise indicated. Measurements were conducted prior to dialysis.

Abbreviations: BMI, body mass index; BP, blood pressure; eGFR, estimated glomerular filtration rate; Kt/V, *K*– clearance of urea, *t*– time, *V*– volume of distribution of urea, approximately equal to patient's total body water; nPCR, normalized protein catabolic rate; PEW, protein-energy wasting; SGA, Subjective Global Assessment.

a Median (interquartile range).

b The residual eGFR was based on combined urea and creatinine clearance and corrected for body surface.

c Mean systolic and diastolic BP values shown are prior to dialysis, as measured over the previous 2 weeks.

d Cardiovascular disease was defined as any history of a cerebral vascular accident, a myocardial infarction peripheral vascular disease, or heart failure.

e The nPCR was calculated using the Watson nomogram.

### Mortality Risk

During 10 years of follow-up, 75 (7%) patients receiving hemodialysis switched to peritoneal dialysis and were thus censored. Median survival for all patients during 10 years of follow-up was 3.93 years. In total, 555 deaths were observed over 2,915 person-years, resulting in an overall crude all-cause mortality rate of 19.0 per 100 patient-years (95% CI, 17.5-20.7 per 100 patient-years). Approximately 29% of all deaths were due to cardiac causes. The Kaplan-Meier curves showing the survival probabilities for each baseline predialysis serum potassium level category during 10 years of follow-up are demonstrated in Figure S1. Table 2 shows the mortality rates (95% CIs) per 100 patient-years during 10 years of follow-up according to the 6 time-dependent potassium level categories. The absolute risk of death was clearly increased in the lowest predialysis serum potassium level category of ≤4.0 mmol/L compared with >5.0 to ≤5.5 mmol/L, which corresponded to an excess rate of approximately 9 deaths/100 patient-years. The highest predialysis serum potassium level category of >6.0 mmol/L corresponded to an excess rate of approximately 2 deaths/100 patient-years compared with the predialysis serum potassium level category of >5.0 to ≤5.5 mmol/L.

**Table 2:** Absolute All-Cause Mortality Rates (95% Confidence Intervals) According to 6 Time-Dependent Predialysis Serum Potassium Level Categories During 10-Year Follow-up of 1,117 Incident Hemodialysis Patients

All-Cause Mortality	Time-Dependent Predialysis Serum Potassium Level Category, mmol/L					
	≤4.0	>4.0 to ≤4.5	>4.5 to ≤5.0	>5.0 to ≤5.5	>5.5 to ≤6.0	>6.0
Person-years	199.5	451.7	722.1	712.2	506.2	323.3
Deaths from all causes	52	86	154	122	78	63
Deaths from all causes/100 person-years	26.1 (19.0-33.1)	19.0 (15.0-23.1)	21.3 (17.9-24.7)	17.1 (14.1-20.2)	15.4 (12.0-18.8)	19.5 (14.7-24.3)

After checking the proportional hazards assumption, we found no sign of violation. Table 3 shows the crude and multivariable adjusted relationship between the 6 time-dependent predialysis serum potassium level categories and 6-month mortality during 10 years of follow-up. Additional adjustments for residual kidney function or SGA did not materially attenuate the relationship between predialysis serum potassium level and mortality. The HR of time-dependent predialysis serum potassium ≤4.0 mmol/L was 1.42 (95% CI, 1.01-1.99), implying that hypokalemia is a 1.4-fold stronger risk factor compared with predialysis serum potassium levels of >5.0 and ≤5.5 mmol/L, whereas predialysis serum potassium levels of >6.0 mmol/L resulted in an HR of 1.32 (95% CI, 0.97-1.81).

**Table 3:** Hazard Ratios With 95% Confidence Intervals of 6-Month All-Cause Mortality According to the 6 Categories of Time-Dependent Predialysis Serum Potassium Levels in 1,117 Incident Hemodialysis Patients During 10 Years of Follow-up

Time-Dependent Predialysis Serum Potassium Level Category, mmol/L	Hazard Ratio (95% CI)				
	Crude	Age and Sex Adjusted	Model 1	Model 2	Model 3
≤4.0	1.64 (1.18-2.29)	1.47 (1.05-2.05)	1.40 (1.00-1.96)	1.48 (1.05-2.07)	1.42 (1.01-1.99)
>4.0 to ≤4.5	1.22 (0.92-1.62)	1.13 (0.85-1.50)	1.04 (0.78-1.38)	1.10 (0.82-1.46)	1.09 (0.82-1.45)
>4.5 to ≤5.0	1.28 (1.00-1.63)	1.18 (0.92-1.51)	1.17 (0.91-1.50)	1.22 (0.95-1.57)	1.21 (0.94-1.56)
>5.0 to ≤5.5	1	1	1	1	1
>5.5 to ≤6.0	0.94 (0.70-1.26)	0.96 (0.72-1.29)	0.98 (0.73-1.31)	0.96 (0.71-1.29)	0.95 (0.71-1.28)
>6.0	1.23 (0.90-1.69)	1.39 (1.02-1.90)	1.38 (1.01-1.89)	1.36 (0.99-1.85)	1.32 (0.97-1.81)

*Note:* The serum potassium level of 5.0 to 5.5 mmol/L was taken as the reference category. Model 1 was adjusted for age, sex, current smoking, history of diabetes mellitus, and history of cardiovascular disease. Model 2 had an additional adjustment for residual kidney function. Model 3 (full model) had an additional adjustment for residual kidney function and subjective global assessment score.

Abbreviation: CI, confidence interval.

### U-Shaped Relationship

**Figure 1:**
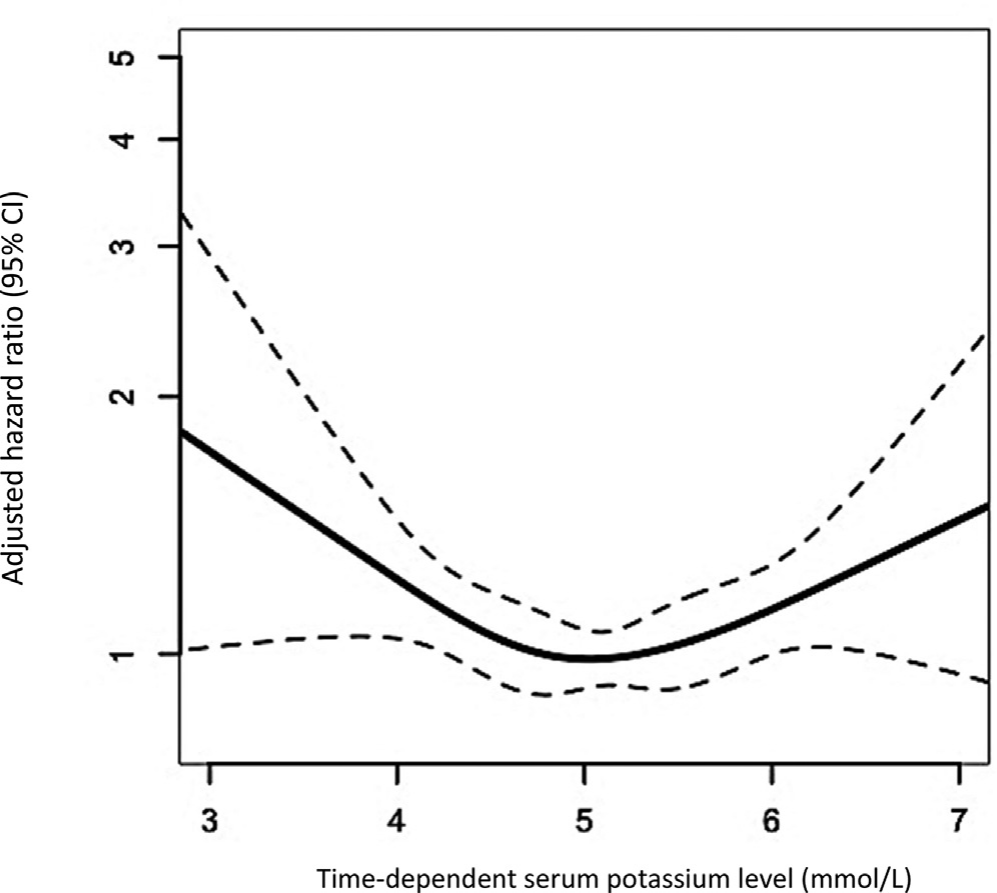
Hazard ratio for 6-month all-cause mortality with 95% confidence intervals (dotted lines) related to time-dependent predialysis serum potassium levels in 1,117 incident hemodialysis patients during 10 years of follow-up, calculated from our 4-knot restricted cubic spline and adjusted for age, sex, current smoking, a history of diabetes mellitus, and a history of cardiovascular disease.

Figure 1 shows the U-shaped relationship between time-dependent predialysis serum potassium level and 6-month mortality during 10 years of follow-up, expressed using the multivariable adjusted HR, with a nadir at approximately 5.1 mmol/L. HRs for death increased substantially below a predialysis serum potassium level of ≤4.5 mmol/L and above >5.7 mmol/L, with the effects of lower predialysis serum potassium levels being more pronounced. For example, patients receiving hemodialysis with a predialysis serum potassium level of 4.0 mmol/L compared with the optimum level of 5.1 mmol/L had an almost 1.4-fold increased risk of death.

### Sensitivity Analyses

We performed 4 sensitivity analyses. First, additional adjustments for the nutritional markers phosphate, bicarbonate, albumin, and normalized protein catabolic rate did not materially alter the results of our main analysis (Table S1). Second, considering cardiac death as an outcome attenuated the results due to the loss of power but showed a similar direction of the relationship (Table S2). Third, repeating our analyses without censoring for patients who switched to peritoneal dialysis also did not substantially change the effect between predialysis serum potassium level and death (Table S3). Finally, considering predialysis serum potassium level as a fixed category at baseline did attenuate the strength of the relationship between predialysis serum potassium level category and 10-year all-cause mortality as expected due to the dilution of the effect of potassium over time (Tables S4 and S5).

## Discussion

This prospective study among exclusively incident hemodialysis patients showed a U-shaped relationship between predialysis serum potassium level and death during 10 years of follow-up, with an optimum level of approximately 5.1 mmol/L. Compared with this optimum level, low (≤4 mmol/L) and high (>6 mmol/L) predialysis serum potassium concentrations were 1.4- and 1.3-fold stronger risk factors for death after multivariable adjustment, respectively.

Our results are in line with the study by Torlén et al,^3^ including >1,11,000 prevalent hemodialysis patients showing adjusted HRs for all-cause mortality for time-dependent serum potassium levels of <3.5 mmol/L and ≥5.5 mmol/L of 2.0 (95% CI, 1.8-2.1) and 1.2 (95% CI, 1.2-1.3) compared with the reference category of 4.0-4.5 mmol/L, respectively. A limitation of this previous study is the inclusion of prevalent patients, which may have resulted in selection bias. In addition, estimates were not adjusted for the potential confounders residual kidney function and nutritional status. In contrast, another study including 55,000 prevalent hemodialysis patients showed low adjusted HRs for death of 1.0 (95% CI, 1.0-1.1) and 1.1 (95% CI, 1.0-1.2) for serum potassium <4.0 mmol/L and >6.0 mmol/L, respectively, compared to the reference category of 4.0 to 5.0 mmol/L.^12^ Next to selection bias and their relatively low reference category, another possible explanation for their low HRs could be the use of serum potassium as a fixed variable in their model over a median follow-up of 16.5 months. When considering predialysis serum potassium level as a fixed value at baseline, we had a similar finding, namely, a weakening of the observed effect over time most likely due to dilution.

The relatively high optimum predialysis serum potassium level of 5.1 mmol/L that we found is consistent with 2 previous studies. Kovesdy et al^19^ reported that predialysis serum potassium levels between 4.6 and 5.3 mmol/L were associated with the highest 3-year survival rate in >81,000 prevalent hemodialysis patients. Pun et al^20^ found that a predialysis serum potassium level of 5.1 mmol/L resulted in the lowest risk of sudden cardiac arrest.

Limitations of these 2 previous studies were again the inclusion of prevalent patients and, in the study by Pun et al,^20^ failure to adjust for potential confounders such as smoking, residual kidney function, and nutritional status.

Serum potassium concentration rapidly decreases by approximately 1 mEq/L in the first hour of hemodialysis treatment, when the blood-to-dialysate gradient is greatest, and an additional lowering of 1 mEq/L occurs over the next 2 hours. A rapid rebound of serum potassium occurs because of efflux from the intracellular compartment after completion of the dialysis session.^7^ Patients with a relatively low predialysis serum potassium level may experience more severe or prolonged hypokalemia after the session, which may explain the increased risk of death that we found.^21^ Another explanation could be that low predialysis serum potassium level is a proxy for malnourishment, which is a strong risk factor for death.^22^^,^^23^ However, adjusting for SGA score, the normalized protein catabolic rate, serum phosphate, bicarbonate, and albumin, as indicators of malnutrition, did not materially attenuate the relationship between predialysis serum potassium level and death. Finally, low total body potassium is also a risk factor for death.^8^ Hemodialysis treatment may reduce total body potassium by depleting intracellular potassium stores. Although serum potassium level does not necessarily reflect the intracellular potassium, a low predialysis concentration may serve as a proxy of a relatively low total body potassium level.

There are several strengths to our study. First, to our knowledge, this is the first study that included only incident hemodialysis patients. All previous studies investigating the relationship between predialysis serum potassium level and death mainly included prevalent hemodialysis patients, thus being susceptible to survivor bias.^3^^,^^4^^,^^11^^,^^12^^,^^19^^,^^23^^,^^24^ This is a form of selection bias that occurs when the risk of an outcome is estimated from data collected at a given time point among survivors rather than on data gathered in a group of incident cases.^10^ As with other biases, an increased study size cannot compensate for survivor bias.^25^ Second, all measurements were performed according to the study protocol, and, therefore, information bias is unlikely. This is in contrast to the previous studies in which data were collected from clinical records, potentially resulting in information bias as data were collected for a clinical reason. Third, we adjusted for smoking, an important confounder that was unavailable in the majority of the previous studies and, therefore, not included in the model.^4^^,^^11^^,^^12^^,^^23^^,^^24^ Reverse causation owing to inadequate control for smoking status can distort the true relationship between hypokalemia and the risk of death because smoking is associated with both decreased serum potassium level and an increased risk of death.^26^ Finally, by modeling potassium freely in our restricted cubic spline analysis, we could establish the optimum predialysis serum potassium level as a reference category and incorporate it into our main time-dependent analysis. As this reference was found to be higher than those used in previous studies, this may have allowed for a more valid estimation of the HRs associated with dyskalemia, particularly hypokalemia. Using a lower reference category, as most previous studies did, could have resulted in the underestimation of the relative relationship between hypokalemia and mortality.

Nevertheless, our study has some limitations. First, as with most studies, we encountered missing data. To maintain power and minimize any bias, we used multiple imputations to account for these missing data. Second, even though we updated predialysis serum potassium level as a time-dependent variable, we could only do so for every 6 months. As the serum predialysis potassium level fluctuates, shorter intervals between measurements would have allowed for more precise estimations of the mortality risk and less “dilution” of the effect. Third, as we did not have information on peridialytic changes in serum potassium levels, the serum-to-dialysate potassium gradient, or postdialysis serum potassium levels, we could not consider the effects of these factors on the mortality risk. Fourth, we used all-cause mortality as the primary outcome, which is unequivocal, whereas the secondary outcome, cardiac death, can be nondifferentially misclassified. Considering cardiac death as an outcome showed slightly weaker but similar results. In general, nondifferential misclassification results in an underestimation of the effect.^25^

In conclusion, we found a U-shaped relationship between predialysis serum potassium levels and 6-month all-cause mortality in incident hemodialysis patients in the first 10 years of follow-up. Our results indicate an optimum predialysis serum potassium level of approximately 5.1 mmol/L. Low and high predialysis serum potassium levels resulted in 1.4- and 1.3-fold stronger risk factors for death, respectively, compared with the optimum level. If proven causal, the clinical implication of these results is that potassium-lowering therapy should be used with caution in patients receiving hemodialysis with normal or low serum potassium levels before the dialysis session. Furthermore, as low predialysis serum potassium level could result from malnourishment, the associated mortality risk emphasizes the importance of preventing nutritional disorders in patients receiving hemodialysis.
