# Supplementary material for: Serum Potassium and Mortality Risk in Hemodialysis Patients: A Cohort Study
Source: Kidney Med. 2021 Oct 22;4(1):100379. doi: 10.1016/j.xkme.2021.08.013 (PMC8767120; doi:10.1016/j.xkme.2021.08.013)
Supplement: Supplementary File (PDF) — Figure S1, Table S1-S5. [file mmc1.pdf]

Figure S1. Kaplan-Meier curves showing the survival probability for each baseline serum potassium category over 10 years of follow-up

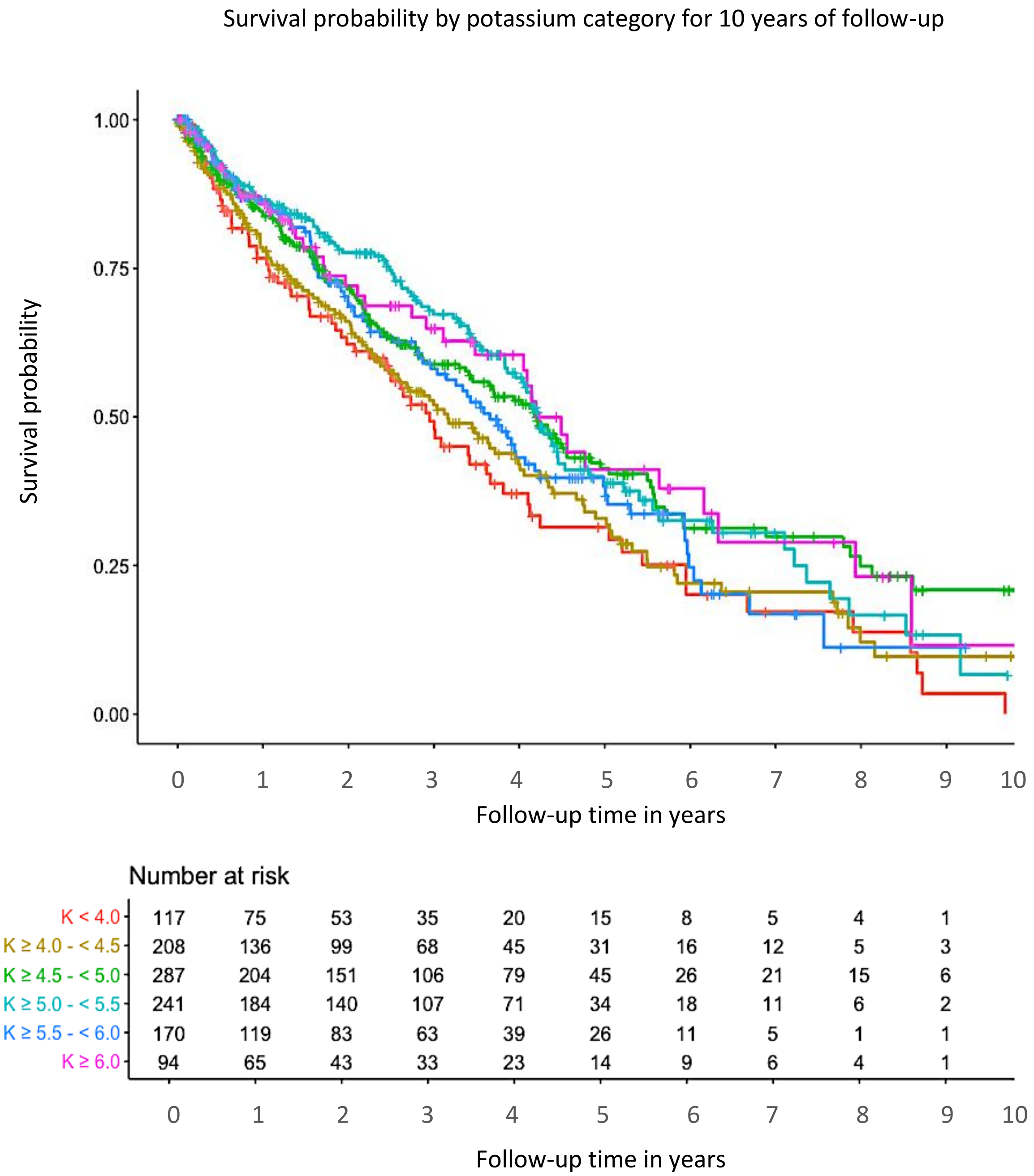

TABLE S1. Hazard ratios with 95% confidence intervals of 6-month all-cause mortality according the six categories of time-dependent predialysis serum potassium in 1117 incident hemodialysis patients during 10 years of follow-up with additional adjustment for nutritional markers

Time-dependent predialysis serum potassium category (mmol/L)	Hazard Ratio (95% CI)					
	Full model*	Full model adjusted for serum phosphate	Full model adjusted for serum bicarbonate	Full model adjusted for Serum albumin	Full model adjusted for nPCR	Full model adjusted for all**
≤ 4.0	1.42 (1.01-1.99)	1.44 (1.02-2.02)	1.42 (1.01-1.99)	1.42 (1.01-1.99)	1.40 (1.00-1.97)	1.43 (1.02-2.01)
> 4.0 to ≤ 4.5	1.09 (0.82-1.45)	1.09 (0.82-1.45)	1.09 (0.82-1.45)	1.08 (0.81-1.44)	1.09 (0.81-1.45)	1.09 (0.81-1.45)
> 4.5 to ≤ 5.0	1.21 (0.94-1.56)	1.21 (0.94-1.56)	1.21 (0.94-1.56)	1.21 (0.94-1.56)	1.21 (0.94-1.55)	1.22 (0.95-1.56)
> 5.0 to ≤ 5.5	1	1	1	1	1	1
> 5.5 to ≤ 6.0	0.95 (0.71-1.28)	0.95 (0.71-1.28)	0.95 (0.71-1.28)	0.95 (0.71-1.28)	0.96 (0.71-1.28)	0.95 (0.71-1.28)
> 6.0	1.32 (0.97-1.81)	1.31 (0.96-1.80)	1.32 (0.96-1.80)	1.33 (0.97-1.83)	1.33 (0.97-1.83)	1.34 (0.98-1.83)

Serum potassium level 5.0 to 5.5 mmol/L was taken as the reference category. nPCR: normalized protein catabolic rate.
*Full model: Adjusted for age, sex, current smoking, history of diabetes mellitus, history of cardiovascular disease, residual kidney function and subjective global assessment score
**Full model with additional adjustment for serum phosphate, serum bicarbonate, serum albumin and nPCR

TABLE S2. Hazard ratios with 95% confidence intervals of 6-month cardiac death according the six categories of time-dependent predialysis serum potassium in 1117 incident hemodialysis patients during 10 years of follow-up

Time-dependent predialysis serum potassium category (mmol/L)	Hazard Ratio (95% CI)				
	Crude	Age and sex adjusted	Model 1	Model 2	Model 3
≤ 4.0	1.31 (0.69-2.49)	1.21 (0.64-2.29)	1.17 (0.62-2.23)	1.17 (0.62-2.24)	1.15 (0.60-2.20)
> 4.0 to ≤ 4.5	1.27 (0.78-2.08)	1.20 (0.73-1.97)	1.11 (0.68-1.82)	1.11 (0.68-1.83)	1.11 (0.68-1.84)
> 4.5 to ≤ 5.0	1.02 (0.64-1.62)	0.95 (0.60-1.51)	0.95 (0.60-1.51)	0.95 (0.60-1.51)	0.95 (0.59-1.51)
> 5.0 to ≤ 5.5	1	1	1	1	1
> 5.5 to ≤ 6.0	0.81 (0.47-1.39)	0.83 (0.48-1.43)	0.85 (0.49-1.47)	0.85 (0.49-1.47)	0.85 (0.49-1.47)
> 6.0	0.97 (0.53-1.75)	1.08 (0.60-1.96)	1.08 (0.60-1.96)	1.08 (0.60-1.96)	1.06 (0.59-1.93)

Serum potassium level 5.0 to 5.5 mmol/L was taken as the reference category.
Model 1: Adjusted for age, sex, current smoking, history of diabetes mellitus and history of cardiovascular disease
Model 2: Additional adjustment for residual kidney function
Model 3 (full model): Additional adjustment for residual kidney function and subjective global assessment score

TABLE S3. Hazard ratios with 95% confidence intervals of 6-month all-cause mortality according the six categories of time-dependent predialysis serum potassium in 1117 incident hemodialysis patients over 10 years of follow-up, without censoring at the time of a switch to peritoneal dialysis

Time-dependent predialysis serum potassium category (mmol/L)	Hazard Ratio (95% CI)				
	Crude	Age and sex adjusted	Model 1	Model 2	Model 3
≤ 4.0	1.67 (1.22-2.29)	1.57 (1.15-2.15)	1.51 (1.10-2.07)	1.59 (1.16-2.18)	1.53 (1.11-2.10)
> 4.0 to ≤ 4.5	1.14 (0.87-1.50)	1.08 (0.82-1.43)	1.00 (0.75-1.32)	1.05 (0.79-1.40)	1.05 (0.79-1.40)
> 4.5 to ≤ 5.0	1.25 (0.98-1.60)	1.17 (0.92-1.49)	1.16 (0.91-1.48)	1.21 (0.95-1.55)	1.21 (0.94-1.55)
> 5.0 to ≤ 5.5	1	1	1	1	1
> 5.5 to ≤ 6.0	0.92 (0.69-1.23)	0.95 (0.71-1.26)	0.96 (0.72-1.28)	0.94 (0.70-1.25)	0.94 (0.70-1.26)
> 6.0	1.20 (0.88-1.63)	1.33 (0.98-1.82)	1.32 (0.97-1.80)	1.30 (0.95-1.77)	1.27 (0.93-1.73)

Serum potassium level 5.0 to 5.5 mmol/L was taken as the reference category.
Model 1: Adjusted for age, sex, current smoking, history of diabetes mellitus and history of cardiovascular disease
Model 2: Additional adjustment for residual kidney function
Model 3 (full model): Additional adjustment for residual kidney function and subjective global assessment score

TABLE S4. Hazard ratios with 95% confidence intervals of 10-year all-cause mortality according the six categories of predialysis serum potassium fixed at baseline in 1117 incident hemodialysis patients

	Hazard Ratio (95% CI)				
Baseline predialysis serum potassium category (mmol/L)	Crude	Age and sex adjusted	Model 1	Model 2	Model 3
≤ 4.0	1.58 (1.17-2.13)	1.33 (0.98-1.80)	1.23 (0.91-1.67)	1.27 (0.93-1.73)	1.15 (0.84-1.59)
> 4.0 - ≤ 4.5	1.41 (1.09-1.83)	1.30 (1.00-1.69)	1.26 (0.97-1.64)	1.32 (1.01-1.72)	1.25 (0.95-1.63)
> 4.5 - ≤ 5.0	1.05 (0.81-1.35)	1.01 (0.78-1.29)	1.02 (0.79-1.31)	1.07 (0.83-1.38)	1.07 (0.82-1.38)
> 5.0 - ≤ 5.5	1	1	1	1	1
> 5.5 - ≤ 6.0	1.23 (0.93-1.64)	1.20 (0.90-1.59)	1.21 (0.91-1.62)	1.20 (0.90-1.59)	1.23 (0.92-1.64)
> 6.0	0.99 (0.69-1.43)	1.00 (0.69-1.44)	1.06 (0.73-1.54)	1.01 (0.71-1.47)	0.94 (0.64-1.37)

Serum potassium level 5.0 to 5.5 mmol/L was taken as the reference category.
Model 1: Adjusted for age, sex, current smoking, history of diabetes mellitus and history of cardiovascular disease
Model 2: Additional adjustment for residual kidney function
Model 3 (full model): Additional adjustment for residual kidney function and subjective global assessment score

TABLE S5. Hazard ratios with 95% confidence intervals of 10-year cardiac death according the six categories of predialysis serum potassium fixed at baseline in 1117 incident hemodialysis patients

	Hazard Ratio (95% CI)				
Baseline predialysis serum potassium category (mmol/L)	Crude	Age and sex adjusted	Model 1	Model 2	Model 3
≤ 4.0	2.35 (1.40-3.95)	1.98 (1.17-3.33)	1.85 (1.09-3.14)	1.87 (1.10-3.19)	1.86 (1.08-3.21)
> 4.0 - ≤ 4.5	1.72 (1.06-2.78)	1.59 (0.98-2.57)	1.53 (0.94-2.49)	1.56 (0.96-2.55)	1.54 (0.94-2.52)
> 4.5 - ≤ 5.0	0.91 (0.55-1.51)	0.88 (0.53-1.46)	0.89 (0.54-1.48)	0.91 (0.55-1.52)	0.91 (0.55-1.52)
> 5.0 - ≤ 5.5	1	1	1	1	1
> 5.5 - ≤ 6.0	1.06 (0.60-1.87)	1.02 (0.58-1.81)	1.05 (0.59-1.86)	1.04 (0.59-1.85)	1.05 (0.59-1.87)
> 6.0	0.85 (0.40-1.80)	0.85 (0.40-1.81)	0.93 (0.44-1.97)	0.91(0.43-1.95)	0.88 (0.41-1.88)

Serum potassium level 5.0 to 5.5 mmol/L was taken as the reference category.
Model 1: Adjusted for age, sex, current smoking, history of diabetes mellitus and history of cardiovascular disease
Model 2: Additional adjustment for residual kidney function
Model 3 (full model): Additional adjustment for residual kidney function and subjective global assessment score
